# Decidual natural killer cell receptor expression is altered in pregnancies with impaired vascular remodeling and a higher risk of pre-eclampsia

**DOI:** 10.1189/jlb.2A0614-282R

**Published:** 2014-11-07

**Authors:** Alison E. Wallace, Guy S. Whitley, Baskaran Thilaganathan, Judith E. Cartwright

**Affiliations:** * Institute of Cardiovascular and Cell Sciences, St George’s University of London, United Kingdom; and; † Fetal Medicine Unit, St George’s Hospital, London, United Kingdom

**Keywords:** trophoblast, LILRB1, KIR, chemokine

## Abstract

During pregnancy, a specialized type of NK cell accumulates in the lining of the uterus (decidua) and interacts with semiallogeneic fetal trophoblast cells. dNK cells are functionally and phenotypically distinct from PB NK and are implicated in regulation of trophoblast transformation of the uterine spiral arteries, which if inadequately performed, can result in pregnancy disorders. Here, we have used uterine artery Doppler RI in the first trimester of pregnancy as a proxy measure of the extent of transformation of the spiral arteries to identify pregnancies with a high RI, indicative of impaired spiral artery remodeling. We have used flow cytometry to examine dNK cells isolated from these pregnancies compared with those from pregnancies with a normal RI. We report a reduction in the proportion of dNK cells from high RI pregnancies expressing KIR2DL/S1,3,5 and LILRB1, receptors for HLA-C and HLA-G on trophoblast. Decreased LILRB1 expression in the decidua was examined by receptor blocking in trophoblast coculture and altered dNK expression of the cytokines CXCL10 and TNF-*α*, which regulate trophoblast behavior. These results indicate that dNK cells from high RI pregnancies may display altered interactions with trophoblast via decreased expression of HLA-binding cell-surface receptors, impacting on successful transformation of the uterus for pregnancy.

## Abstract (precis)

HLA-interacting cell surface receptors are altered on decidual natural killer cells in pregnancy, potentially altering interactions with fetal cells via chemokine expression.

## Introduction

During the first trimester of pregnancy, maternal NK cells accumulate in the lining of the pregnant uterus (decidua). dNK cells are functionally and phenotypically distinct from their PB counterparts, and the role they play in pregnancy is still unknown; however, they are implicated in regulation of invasion of the semiallogeneic fetal placenta [1–3]. Disruptions in these interactions have been implicated in the pathology of pregnancy disorders, including pre-eclampsia and recurrent miscarriage [4–7].

The fetal placenta develops from the trophectoderm outer layer of the blastocyst and forms a villous branching structure, from which EVT cells differentiate and invade deeply into the decidua. Within the decidua, EVT remodel the coiled, low-flow, spiral arteries, transforming them into wide-diameter conduits, allowing a greater flow of blood to the fetus [8]. EVTs achieve this through a combination of induced apoptosis and de-differentiation of vascular cells [9–11], eventually replacing the vascular cells they have displaced [12]. To accomplish this aim, EVT must avoid an adverse immune reaction by the dNK cells. This is thought to be achieved partially by the atypical EVT MHC repertoire, as they express the classic polymorphic HLA-C and the nonclassic HLA-E and HLA-G [13]. The unique maternal-fetal immune interaction may additionally be enhanced by the dissimilar phenotype of dNK cells to PB NK cells; dNK cells are predominantly CD56^bright^CD16^–^, noncytotoxic, and cytokine-secreting cells [1]. They also express a different repertoire of inhibitory and activatory receptors to PB NK cells, which includes higher expression of the KIRs KIR2DL1/S1 and KIR2DL2/S2 [14]; the 3 NCRs NKp46, NKp30, and NKp44; and LILRB1 [15].

A failure by EVT to completely remodel spiral arteries and subsequent poor placentation can be an underlying cause of pregnancy disorders, including pre-eclampsia and intrauterine growth restriction [8, 16, 17]. The interaction between dNK and EVT has been implicated in this process [4, 7]. However, the study of first-trimester interactions between dNK cells and EVT and their relationship to disorders of pregnancy are made challenging by a number of factors, including the lack of access to first-trimester human pregnancy tissue with a known outcome at term. Uterine artery Doppler RI, in the first trimester of pregnancy, can be used as a proxy measure of the extent of remodeling of the spiral arteries [18, 19]. We have used this technique to identify pregnancies with a high RI, indicative of impaired spiral artery remodeling, to demonstrate differences in dNK cells isolated from high RI pregnancies in their interactions with vascular cells and trophoblasts compared with dNK cells isolated from pregnancies with a normal RI [7, 20].

Here, we have investigated the receptor expression and cytotoxicity of dNK cells isolated from pregnancies with normal and high RI and the implications of an altered receptor repertoire. We provide evidence that dNK cells from high RI pregnancies may show alterations in their interactions with fetal HLA-C and HLA-G, which may have implications for the regulation of EVT-induced remodeling of the decidua by maternal immune cells.

## MATERIALS AND METHODS

### Doppler ultrasound characterization

Determination of the uterine artery RI was performed in women attending a clinic for termination of pregnancy in the first trimester, as described previously [21] at the Fetal Medicine Unit, St George’s Hospital. The Wandsworth Local Research Ethics Committee approval was in place for the Doppler ultrasound and use of first-trimester tissue after surgical termination, and all women gave informed, written consent (reference numbers: 01.96.8; 01.78.5; 02.6.8). Inclusion criteria were singleton pregnancy, gestational age 9–14 weeks, normal fetal anatomy, and nuchal translucency thickness with no known maternal medical condition or history of recurrent miscarriage. High-resistance cases were defined as those with bilateral uterine diastolic notches and a mean RI above the 95th percentile. Normal resistance cases had no diastolic notches and a mean RI below the 95th percentile. These resistance groups represent cases most (21%) and least (<1%) likely to have developed pre-eclampsia, respectively, had the pregnancy progressed [18, 19].

### dNK cell isolation

dNK cells were isolated, as described previously [7]. In brief, decidual tissue was minced and digested in serum-free M199 media containing 2 mg/ml collagenase and 0.1 mg/ml DNase overnight, with constant agitation at room temperature. The resultant tissue digest was passed sequentially through 100 and 70 *µ*m filters and layered onto Ficoll-Paque (GE Healthcare Life Sciences, Buckinghamshire, United Kingdom). The buffy layer was collected, and cells were resuspended in 10 ml dNK cell-culture media [Phenol Red Free RPMI 1640, supplemented with 10% (v/v) FBS, containing 2 mmol/L L-glutamine, 100 IU/ml penicillin, 100 *µ*g/ml streptomycin, and 2.5 *µ*g/ml amphotericin] and plated in a 37°C incubator for 15 min. Nonadherent cells, containing the dNK cell fraction, were purified by use of negative selection with a MagCellect Human NK Cell Isolation Kit (R&D Systems, Abingdon, United Kingdom), according to the manufacturer’s instructions. Purity, as measured by CD56+ cells, was, on average, 95.7 ± 0.92% (*n* = 33), and viability, immediately upon isolation, was 96.5 ± 0.38% (*n* = 33), as assessed by fixable viability dye (eBioscience, Hatfield, United Kingdom). There was no difference in viability or purity between dNK cells isolated from normal RI or high RI pregnancies. Gestational ages between the two datasets did not differ significantly (normal = 76.4 ± 2.1 days; high = 71.1 ± 1.4 days).

### PB NK cell isolation

PB was taken from healthy volunteers, and PB NK cells, isolated from total mononuclear cells, separated after centrifugation on Ficoll-Paque Plus (GE Healthcare Life Sciences) for 30 min at 400 *g*. PB NK cells were isolated by use of a MagCellect Human NK Cell Isolation kit (R&D Systems), according to the manufacturer’s instructions.

### Cell culture

dNK cells were cultured in dNK culture media as above. K562 cells and sHLA-G-transfected SGHPL-4 were maintained in RPMI 1640 Phenol Red Free, supplemented with 10% (v/v) FBS, containing 2 mmol/L L-glutamine, 100 IU/ml penicillin, and 100 *µ*g/ml streptomycin.

### Flow cytometry

Freshly isolated dNK cells were resuspended in 1 ml PBS and stained with fixable viability dye eFluor 780, according to the manufacturer’s instructions (eBioscience). dNK cells were then washed in FACS buffer (PBS with 0.5% w/v BSA, 0.05% w/v sodium azide) and blocked in 1 *µ*g/ml human IgG. Cells (2 × 10^5^) were resuspended in 100 *µ*l FACS buffer, and cells were labeled by use of the following antibodies: mouse anti-human CD56-Alexa Fluor 488 (B159) 0.5 *µ*g, mouse anti-human CD158b (KIR2DL2/S2)-PE (CH-L) 0.125 *µ*g, mouse anti-human CD69-APC (FN50) 0.015 *µ*g, mouse anti-human NKG2D-APC (1D11) 1 *µ*g, mouse anti-human CD9-PE (M-L13) 0.125 *µ*g, mouse anti-human NKp44-PE (p44-8.1) 0.125 *µ*g, mouse anti-human NKp46-PE (9E2) 0.25 *µ*g, and mouse anti-human NKp30-PE (p30-15) 1 *µ*g (BD PharMingen, Oxford, United Kingdom); mouse anti-human CD3-PerCP (SK-7) 0.125 *µ*g and mouse anti-human KIR2DL1/S1/L3/S3/L5/S5-APC (KIR2DS/L1,3,5; MA4) 0.125 *µ*g (eBioscience); mouse anti-human CD160-PE (688327) 0.25 *µ*g, mouse anti-human NKG2A-APC (131411) 0.1 *µ*g, mouse anti-human LILRB1/ILT2/CD85j-APC (292305) 0.1 *µ*g, and mouse anti-human NKG2C-APC (134591) 0.5 *µ*g (R&D Systems). The following isotype controls were used: mouse IgG1 *κ*-Alexa Fluor 488, mouse IgG2b *κ*-PE, mouse IgG2a *κ*-APC, mouse IgG2b *κ*-APC, mouse IgG1 *κ*-APC, and mouse IgG1 *κ*-PE (eBioscience) and mouse IgG1 *κ*-PerCP (BD PharMingen). Flow cytometry was carried out on a LSR II flow cytometer (BD Biosciences, San Jose, CA, USA). Analysis was carried out by use of FlowJo software (Tree Star, Ashland, OR, USA). Histograms shown were gated on viable cells, which were CD56^+^CD3^–^.

### Cytotoxicity assay

Cytotoxicity of NK cells was assessed by lysis of K562 target cells, loaded with the fluorescent dye calcein-AM (Life Technologies, Paisley, United Kingdom). K562 cells were incubated and loaded with 10 *µ*M calcein-AM for 30 min at 37°C, before washing in K562 maintenance media and serum-free media for 15 min. K562 cells and freshly isolated dNK cells or PB NK cells were cocultured in serum-free dNK culture media in V-bottom, 96-well plates (Corning Life Sciences, The Netherlands) for 4 h at ratios of 1:1–20:1 E:T. Calcein-AM released into the supernatant was assessed by use of a GloMax-Multi+ microplate spectrofluorimeter (Promega, Southampton, United Kingdom) with excitation filter 485 and emission filter 530. Data are expressed as fold lysis over control, containing no NK cells but matched numbers of target cells.

### LILRB1 blocking

Receptor blocking was achieved by incubating freshly isolated dNK cells for 30 min at 37°C with 10 *µ*g/ml mouse anti-human LILRB1/ILT2/CD85j mAb (clone 292319; R&D Systems) or isotype-matched control (R&D Systems) [22] before coculture in media containing 10 *µ*g/ml mouse anti-human LILRB1 with SGHPL-4 cells overexpressing sHLA-G [23] for 6 h.

### PCR

Cytokine expression in dNK cells was assessed by RT-PCR. dNK RNA samples (*n* = 6) were reverse transcribed by use of the Tetro cDNA Synthesis kit, according to the manufacturer’s instructions (Bioline, London, United Kingdom). cDNA (40 ng) was used in duplicate samples for qRT-PCR by use of Power SYBR Green PCR Master Mix (Applied Biosystems, Life Technologies, Pittsburgh, PA, USA), as per the manufacturer’s instructions, by use of the following sequence-specific primers: 18S, ACA-CGT-TCC-ACC-TCA-TCC-TC and CTT-TGC-CAT-CAC-TGC-CAT-TA; CXCL10, TTC-AAG-GAG-TAC-CTC-TCT-CTA-G and CTG-GAT-TCA-GAC-ATC-TCT-TCT-C; PLGF, GTC-TCC-TCC-TTT-CCG-GCT-T and TGC-AGC-TCC-TAA-AGA-TCC-GTT; IFN-*γ*, ACT-GAC-TTG-AAT-GTC-CAA-CGC-A and ATC-TGA-CTC-CTT-TTT-CGC-TTC-C; IL-8, CAG-AGA-CAG-CAG-CAC-AC and AGC-TTG-GAA-GTC-ATG-TTT-ACA-C; TNF, AGG-TTC-TCT-TCC-TCT-CAC-ATA-C and ATC-ATG-CTT-TCA-GTG-CTC-ATG. qPCR was carried out by use of a CFX96 Real-Time PCR Detection System (Bio-Rad Laboratories, Hemel Hempstead, United Kingdom). Expression of analyzed genes was normalized to RNA loading for each sample by use of the 18S rRNA as an internal standard, and each LILRB1-blocked sample was compared with isotype-matched control.

### Statistical analysis

Where appropriate, data were analyzed by one-way ANOVA or Student's *t*-test by use of GraphPad Prism (v6.01; GraphPad Software, La Jolla, CA, USA). Data are presented as mean ± sem.

## RESULTS

### Receptor repertoire of dNK cells isolated from high RI differs from dNK cells isolated from normal RI pregnancies

**Figure 1.**
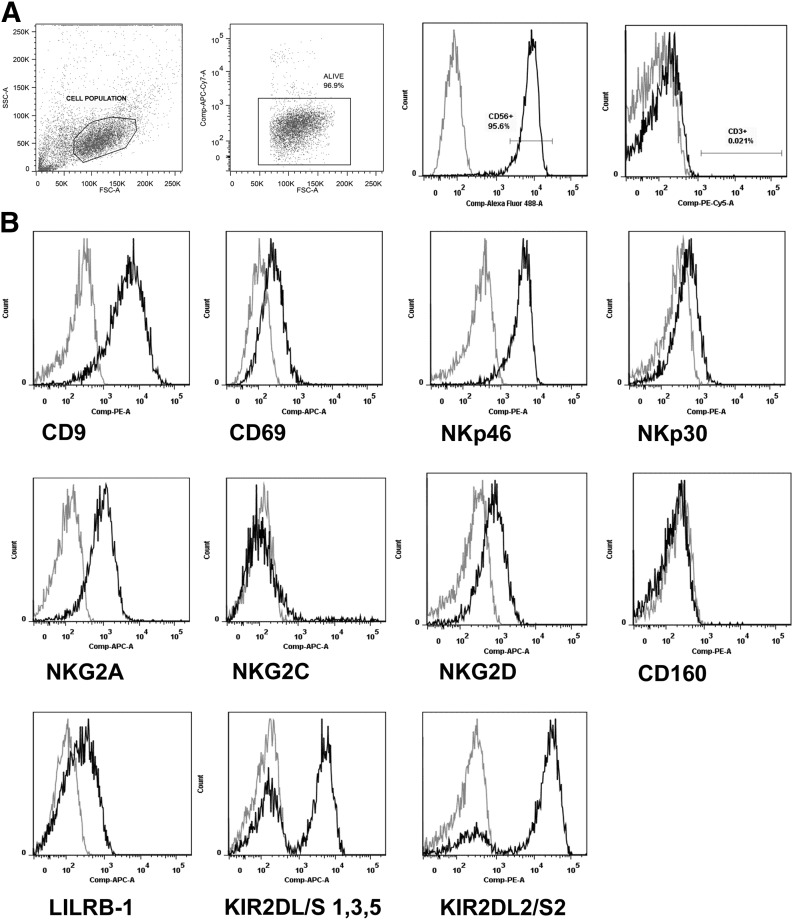
Representative flow cytometry data of cell-surface receptor expression on first-trimester dNK cells. (A) Gating strategy. Cell population was automatically gated on forward (FSC)/side-scatter (SSC). This population was gated further as dNK cells on viability as assessed by negativity for eFluor dye, CD56 positivity and CD3 negativity. (B) Typical dNKR expression. Data are of a normal RI sample, gestational age 9 + 0 weeks. Compensated (Comp) fluorescence intensity for the gated area is shown. Gray line indicates IgG control, and darker line indicates test antibody to stated receptor.

**Figure 2.**
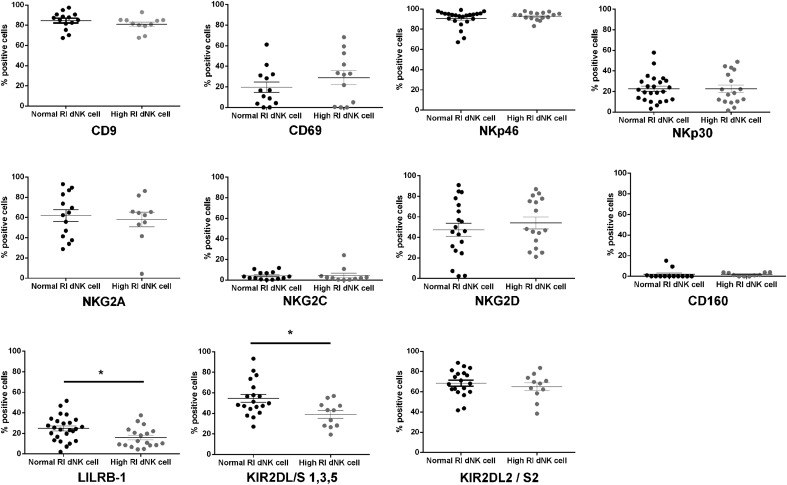
Percentage of dNK cells isolated from normal RI pregnancies and high RI pregnancies positive for receptors listed, as assessed by flow cytometry. Data shown are individual patient samples, mean ± sem; *n* = at least 19 normal RI; *n* = at least 10 high RI. *P < 0.05.

dNK cells have been shown previously to express the following receptors: KIR2DL1/S1, KIR2DL2/S2, NKp30, NKp46, LILRB1, NKG2A, NKG2C, NKG2D, CD160, CD9, and CD69 [14, 15, 24, 25]. Examination of dNK cells (gating strategy; **Fig. 1A**), from normal RI and high RI pregnancies (indicative of poor spiral artery remodeling), determined that these receptors were present in each group (Fig. 1B; representative flow data from normal RI individual). All receptors were expressed in the same proportion in high RI and normal RI pregnancies, with the exception of KIR2DL/S1,3,5 and LILRB1, which were expressed on a significantly lower proportion of dNK cells from high RI pregnancies (*P* < 0.05; **Fig. 2**). Likewise, significantly decreased expression of KIR2DL/S1,3,5 and LILRB1 was found by analysis of mean fluorescence intensity data (*P* < 0.05; Supplemental Fig. 1).

### dNKR repertoire varies with gestational age

**Figure 3.**
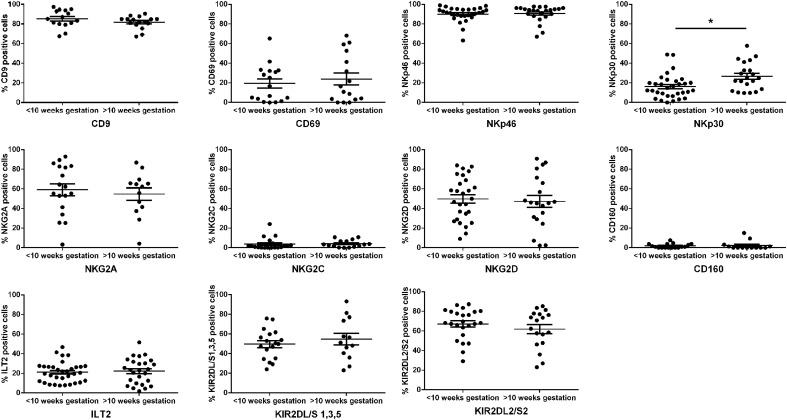
dNKR expression during the first trimester of pregnancy. Percentage expression of the named receptors was analyzed by flow cytometry on dNK cells between 6 and 13 weeks of pregnancy (44–98 days) and separated into before and after 10 weeks gestation. Data shown are mean ± sem; *P < 0.05; *n* = at least 33 in each group.

Percentages of dNK cells expressing receptors, including KIR2DL1/S1, LILRB1, and NKG2D, have been demonstrated to alter throughout the first trimester of pregnancy [26, 27]. The function of dNK cells has also been demonstrated to alter between early gestation and after loosening of trophoblast plugs of spiral arteries, which occurs at ∼10 weeks gestation, for example, in secreted cytokines and interactions with trophoblast [28, 29]. Therefore, we examined the expression of KIR2DL/S1,3,5, KIR2DL2/S2, NKp30, NKp46, LILRB1, NKG2A, NKG2C, NKG2D, CD160, and CD69 in the first trimester of pregnancy, before and after 10 weeks of gestation (44–98 gestational days, separated into <10 weeks or >10 weeks; *n* = at least 33). To eliminate any confounding factors of decreased expression of KIR2DL/S1,3,5 and LILRB1 on high RI cells, these were excluded from the analysis. We found that the majority of receptors did not alter in numbers of dNK cells with gestational age (**Fig. 3**). Expression of NKp30 increased as gestational age increased (*P* = 0.01).

### dNK cells from normal RI and high RI pregnancies are not cytotoxic

**Figure 4.**
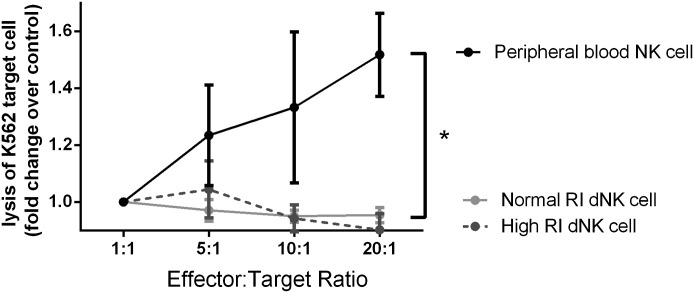
Cytotoxicity of dNK cells from normal RI and high RI pregnancies. The target cell K562 was loaded with fluorescent dye, and the ability of dNK cells from normal RI and high RI pregnancies to lyse target cells was compared with the lytic capacity of PB NK cells. *P < 0.05, data shown are mean ± sem expressed over control containing no effector cells (*n* = 3).

dNK cells are not thought to be cytotoxic in vivo. To determine if any differences in receptor phenotype altered the cytotoxic capacity of dNK cells, the target cell K562 was loaded with fluorescent dye. The ability of dNK cells from normal RI and high RI pregnancies to lyse target cells was compared with the lytic capacity of PB NK cells used as a technical control (**Fig. 4**). dNK showed no significant lytic ability over a control containing no effector cells and was significantly less cytotoxic than PB NK cells (*P* < 0.05).

### dNK cells with lowered LILRB1 binding capacity demonstrate altered cytokine production

**Figure 5.**
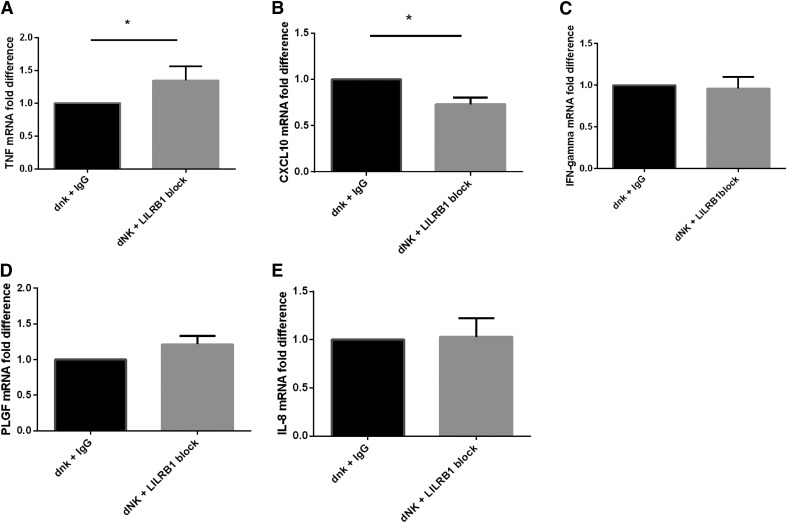
Cytokine mRNA expression in normal RI dNK cells in coculture with SGHPL-4 cells after blocking of LILRB1. Normal RI dNK cells were cocultured with sHLA-G-SGHPL-4 cells for 6 h and collected. Cytokine expression was analyzed by qRT-PCR of (A) TNF, (B) CXCL10, (C) IFN-*γ*, (D) PLGF, (E) IL-8. Data shown are mean fold change ± sem relative to IgG control. *P < 0.05; *n* = 6.

A decrease in expression of LILRB1 may lead to a decreased capacity to bind ligand on trophoblast. To determine if this altered dNK cell activity, dNK cells from normal RI pregnancies (to ensure a larger proportion of LILRB1-expressing dNK cells) were cocultured with an EVT cell line overexpressing HLA-G [23], and the LILRB1 blocked with a blocking antibody. Cytokine production in dNK cells was measured by PCR (**Fig. 5**). Expression of TNF-*α* was found to be increased in dNK cells with decreased LILRB1 binding capacity (Fig. 5A; *P* < 0.05), and expression of CXCL10 was found to be decreased (Fig. 5B; *P* < 0.05). Expression of three other cytokines shown to be important in dNK-trophoblast interactions—IFN-*γ*, PLGF, and IL-8—did not alter (Fig. 5C–E).

## DISCUSSION

During the first trimester of pregnancy, dNK cells interact with fetal trophoblast through secreted factors and cell–cell interactions [3]. The pattern of inhibitory and activating receptors on dNK cells is distinct to that of PB NK and is thought to be crucial in the interaction with fetal trophoblast [30]. We have demonstrated that the levels of HLA-interacting receptors, LILRB1 and KIR2DL/S1,3,5, are reduced on populations of dNK cells from pregnancies with a high RI, indicative of poor spiral artery remodeling. This has implications for interactions of dNK with trophoblast in these pregnancies and in the development of the pathology of pregnancy disorders, such as pre-eclampsia.

A number of receptors were expressed on equal proportions of dNK cells from normal RI and high RI pregnancies. This includes NKp46, an NCR that has been shown to lead to cytotoxic granule release upon engagement, which is negatively regulated by NKG2A [24]. A second natural cytoxicity receptor, NKp30, was also expressed; however, engagement of this on dNK cells has been demonstrated previously to be noncytotoxic and induces cytokine production, including TNF-*α* and GM-CSF [15]. NKp30 is not found in endometrial NK cells from nonpregnant individuals, and this corresponds with our finding that this receptor is expressed on increasing proportions of dNK cells as gestation increases [31]. High expression of CD9 and a mean expression of 19–28% of CD69 are also consistent with previous reports of dNK cell phenotype [24, 26, 27] as is the low or absent expression of CD160 reported [15], and the varied levels of NKG2A [26, 32]. We found comparable expression of NKG2D in dNK cells from normal RI and high RI pregnancies. NKG2D, a NCR, has been implicated in cytokine production [33] and may be involved in cytotoxicity in certain situations, including viral infection [34].

The decreased expression of KIR2DL/S1,3,5 is intriguing, as this has implications for recognition of fetal HLA-C. It has been found that mothers with a KIR-A genotype are at increased risk of pregnancy disorders, including pre-eclampsia and recurrent miscarriage, particularly when paired with a fetus with a HLA-C2 genotype [4–6]. The KIR genotype of the mother determines expression of KIRs; there are two forms of the KIR2DL1/S1 receptor: the long cytoplasmic-tailed inhibitory form L1 and the short cytoplasmic-tailed activatory form S1. A decrease in the S1 form may lead to less cytokine secretion by dNK cells, as dNK cells coexpressing L1 or expressing L1 alone demonstrate dramatic reduction in secretion of cytokines, potentially leading to decreased trophoblast invasion and spiral artery remodeling [35]. As KIR-A mothers have less activating KIRs, this reduced cytokine production and hence, decreased trophoblast invasion are proposed to underlie the association with pregnancy disorders. During preparation of this manuscript, the antibody clone used to distinguish KIR2DL1/S1 was found to recognize L3/S3 and L5/S5 also. Therefore, it will be interesting in future investigations to determine whether expression of S1 or L1 or both is decreased in the populations of high RI dNK cells and additionally, the HLA-C status of the fetus. We have previously demonstrated altered, secreted factors in the high RI dNK cell population, resulting in decreased trophoblast chemotaxis and explant outgrowth [7]; however, whether the finding in this study of altered KIR2DL1/S1 expression on these two groups is connected to decreased cytokine secretion and trophoblast chemotaxis remains to be determined. Interestingly, KIR2DL2/S2 was not found to be decreased in the population of dNK cells in high RI pregnancies. KIR2DL2/S2 binds with a higher affinity to HLA-C1, as opposed to HLA-C2, and whether it has a role in pregnancy disorders remains to be determined.

We have also demonstrated a decreased proportion of LILRB1-expressing dNK cells in the group of pregnancies with a high RI. LILRB1 is an inhibitory receptor that binds to a wide spectrum of HLA molecules but preferentially, to HLA-G [36] and has been demonstrated to bind to HLA-G, expressed on trophoblast [37], and to sHLA-G [22]. The restricted expression of HLA-G in the body to fetal trophoblast indicates that it may have important functions in pregnancy. HLA-G-LILRB1 binding has been demonstrated previously to alter cytokine secretion in decidual leukocytes [37], as well as inhibit formation of the cytotoxic immune synapse [38]. A decreased proportion of dNK cells expressing this receptor could indicate altered immune interactions at the maternal–fetal interface. Therefore, we used an EVT cell line expressing HLA-G and sHLA-G [23], cocultured with dNK cells, and blocked LILRB1 to model the outcome of decreased signaling via this mechanism in the high RI group. The blocking of LILRB1 via this mechanism has been demonstrated previously in PB NK cells [22] and the NK-92 cell line [39]. We found increased expression of TNF-*α* and decreased expression of CXCL10 in the LILRB1-blocked group, although at low levels, which may be representative of the small subset of cells expressing this receptor. This is in contrast to Li et al. [40], who did not demonstrate altered TNF-*α* expression by dNK cells upon blocking of LILRB1; however, this may be reflective of the cell types used for coculture or assays. Similarly to the findings of others, we found no difference in gene expression of IFN-*γ* or IL-8 [41] or of PLGF after blocking LILRB1. A decrease in CXCL10 expression upon interaction with EVT could indicate the potential for decreased induction of EVT migration [2]. Similarly increased TNF-*α* signaling could inhibit EVT invasion [42, 43] and integration of EVT into decidual spiral arteries [44] and induce EVT apoptosis [42]. These features could impact on vessel transformation in high RI pregnancies.

The decrease in LILRB1 and KIR2DL/S1,3,5-expressing dNK cells is particularly interesting, as these receptors interact with the unusual MHC repertoire of the EVT. Despite this decrease in inhibitory receptors in the high RI group, we found no difference in cytotoxicity between the two groups. dNK cells have been shown previously to be cytotoxic only in the presence of nonphysiologic IL-2 stimulation [45], and we found that dNK cells isolated from normal RI and high RI pregnancies exhibited a similarly low level of cytotoxicity to the classic target cell K562 compared with PB NK cells, indicating any difference in cell-surface receptor phenotype was not altering cytotoxicity. Increased expression of LILRB1 is more common on cells with increased KIR2DL1/S1 [41], and therefore, it may be a subset of cells that is decreased in the high RI group. As interactions with KIRs and LILRB1 in dNK cells have been demonstrated to be important in cytokine secretion, it may be that a decrease in this subset of cells leads to an overall suppression of dNK-induced trophoblast invasion and therefore, transformation of spiral arteries. Further investigation into the phenotype of dNK cells from this high-risk group of pregnancies will provide additional insights into maternal-mediated effects on spiral artery remodeling in the first trimester of pregnancy.

## Abbreviations

AM: acetoxymethyl ester
APC: allophycocyanin
CD: cluster of differentiation
dNK: decidual NK
EVT: extravillous trophoblast
ILT2: Ig-like transcript 2
KIR: killer Ig-like receptors
LILRB1: leukocyte Ig-like receptor subfamily B member 1
NCR: natural cytotoxicity receptor
PB: peripheral blood
PLGF: placental growth factor
qRT-PCR: quantitative RT-PCR
RI: resistance index
sHLA: soluble HLA
